# Single‐cell transcriptomics identify TNFRSF1B as a novel T‐cell exhaustion marker for ovarian cancer

**DOI:** 10.1002/ctm2.1416

**Published:** 2023-09-15

**Authors:** Yan Gao, Hui Shi, Hongyu Zhao, Mengcheng Yao, Yue He, Mei Jiang, Jie Li, Zhefeng Li, Shaofei Su, Tao Liu, Chenghong Yin, Xuebin Liao, Wentao Yue

**Affiliations:** ^1^ Central Laboratory Beijing Obstetrics and Gynecology Hospital Capital Medical University Beijing Maternal and Child Health Care Hospital Beijing China; ^2^ School of Pharmaceutical Sciences Beijing Advanced Innovation Center for Human Brain Protection Key Laboratory of Bioorganic Phosphorus Chemistry and Chemical Biology (Ministry of Education) Tsinghua University Beijing China; ^3^ Joint Graduate Program of Peking‐Tsinghua‐NIBS, School of Life Sciences Tsinghua University Beijing China; ^4^ Bioinformatics department Annoroad Gene Technology Co., Ltd Beijing China; ^5^ Department of Gynecology and Oncology Beijing Obstetrics and Gynecology Hospital Capital Medical University Beijing Maternal and Child Health Care Hospital Beijing China; ^6^ Department of Internal Medicine Beijing Obstetrics and Gynecology Hospital Capital Medical University Beijing Maternal and Child Health Care Hospital Beijing China

**Keywords:** Ovarian cancer, single‐cell RNA‐seq, TNFRSF1B, tumour microenvironment

## Abstract

**Background:**

Ovarian cancer (OC) patients routinely show poor immunotherapeutic response due to the complex tumour microenvironment (TME). It is urgent to explore new immunotherapeutic markers.

**Methods:**

Through the single‐cell RNA sequencing (scRNA‐seq) analyses on high‐grade serous OC (HGSOC), moderate severity borderline tumour and matched normal ovary, we identified a novel exhausted T cells subpopulation that related to poor prognosis in OC. Histological staining, multiple immunofluorescences, and flow cytometry were applied to validate some results from scRNA‐seq. Furthermore, a tumour‐bearing mice model was constructed to investigate the effects of TNFRSF1B treatment on tumour growth in vivo.

**Results:**

Highly immunosuppressive TME in HGSOC is displayed compared to moderate severity borderline tumour and matched normal ovary. Subsequently, a novel exhausted subpopulation of CD8^+^TNFRSF1B^+^ T cells is identified, which is associated with poor survival. In vitro experiments demonstrate that TNFRSF1B is specifically upregulated on activated CD8^+^ T cells and suppressed interferon‐γ secretion. The expression of TNFRSF1B on CD8^+^T cells is closely related to OC clinical malignancy and is a marker of poor prognosis through 140 OC patients’ verification. In addition, the blockade of TNFRSF1B inhibits tumour growth via profoundly remodeling the immune microenvironment in the OC mouse model.

**Conclusions:**

Our transcriptomic results analyzed by scRNA‐seq delineate a high‐resolution snapshot of the entire tumour ecosystem of OC TME. The major applications of our findings were an exhausted subpopulation of CD8^+^TNFRSF1B^+^ T cells for predicting OC patient prognosis and the potential therapeutic value of TNFRSF1B. These findings demonstrated the clinical value of TNFRSF1B as a potential immunotherapy target and extended our understanding of factors contributing to immunotherapy failure in OC.

## 1 INTRODUCTION

The tumour microenvironment (TME) is a heterogeneous cellular milieu, that can yet influence tumour progress and impact the effectiveness of treatments.^1^, ^2^ Accumulated evidence indicates that the TME profile plays a role in predicting survival outcomes and evaluating therapeutic efficacy.^3^, ^4^, ^5^ The survival and functional characteristics of tumour cells, and their therapeutic response to treatment strategies, depend on the proportional diversity of the immune cell populations and dysregulation of their respective immune status in the TME.

Tumour‐infiltrating lymphocytes (TILs) play a critical role in the host's immune response within TME. The gene expression profiles of TILs have revealed distinct patterns of immune activation and exhaustion. However different cancer types with similar TIL landscapes exhibit different responses to immunotherapy.^6^ Ovarian cancer (OC) is regarded as a potentially immunoreactive tumour type, with the presence of TILs associated with good clinical outcomes. However, so far, the application of immunotherapy in OC patients has failed to yield clinically meaningful results. Understanding the states and abundances of TILs, which may fundamentally influence drug response to immunotherapies and prognosis, is crucial to developing new efficient immunotherapies for OC.^7^ Based on these findings, the quality and function of TILs, not quantity, may be the deciding factor of immunotherapy response.

Exhausted T cells are considered to be effector T cells with reduced functionality, leading to decreased cytokine secretion and increased expression of inhibitory receptors.^8^ Blockade of these inhibitory molecules, can partially reinvigorate cytotoxicity of T cells and unleash the antitumor response.^9^ However, the effect of programmed cell death 1 (PD‐1) blockade in OCs, has been relatively modest compared to other types of tumors.^10^, ^11^ Taken together, PD‐1 does not completely reflect prognosis and immunotherapy response in OC. Therefore, a more effective exhaustion target for OC is urgently needed to improve patient survival.

Despite advances in the scope of our understanding of intra‐tumour genomic and cellular diversity, the dominant immune cell types and their status in OC remain poorly understood. It thus remains unclear which therapeutic strategies offer the highest potential for the successful targeting of OC tumours. Therefore, we applied scRNA‐seq to characterize the cell subsets in the TME and provide a comprehensive and well‐informed picture of the intercellular factors affecting tumour development and function of TILs. In our study, we found that CD8^+^ T cell exhaustion was the key factor responsible for the immunosuppressive properties. Furthermore, we examined the signature genes of exhausted CD8^+^ T cells in detail, such as TNFRSF1B, and found that highly expressed TNFRSF1B in CD8^+^ T cells could inhabit interferon‐γ (IFN‐γ) production in PBMC, suggesting TNFRSF1B acting as an exhaustion marker and can repress the function of CD8^+^ T cells. Immunostaining of 140 OC tissues demonstrated the highly expressed TNFRSF1B on CD8^+^T cells was closely related to OC clinical malignancy and poor prognosis. Blockade of TNFRSF1B inhibited tumour growth by profoundly remodelling the immune microenvironment in the OC mouse model. Our work identifies TNFRSF1B as a key molecule for T cell exhaustion in OC, targeting TNFRSF1B effectively inhibits OC growth, which can serve as a resource to guide the development of clinical immunotherapies for HGSOC patients.

## 2 RESULTS

### 2.1 Single‐cell profiled the TME of OC and indicated tumour‐specific T cells

**FIGURE 1:**
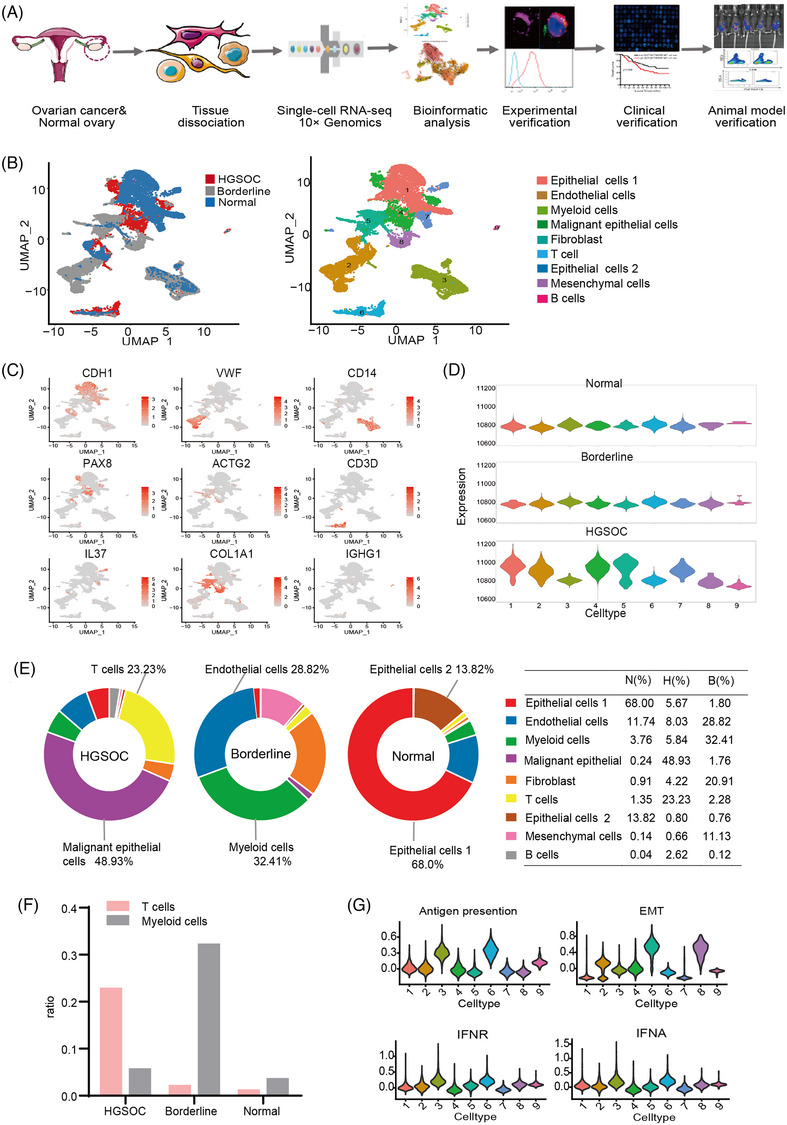
Single‐cell profiled the tumour microenvironment of ovarian cancer (OC) and indicated tumour‐specific T cells. (A) Overview of sample collection and the profiling strategy. (B) Uniform Manifold Approximation and Projection (UMAP) plot of all the single cells, with each colour coded for major cell types and sample origin. (C) Expression levels of specific markers for each cell type are plotted on the UMAP. The colour key from grey to red indicated relative expression levels from low to high. (D)Violin plots showing copy number variation (CNV) levels among nine cell types in different tissues. (E) The ratio of nine cell types in different tissues. Pie charts depicting the two highest cell types in each tissue. (F) The histograms show the ratio of T cells and Myeloid cells in different tissues. (G) Violin plots showing the distribution of the antigen presentation, EMT, interferon (IFN)‐γ response and IFN‐α response signature scores for each cell type.

To explore the cellular diversity of the OC TME and the potential immunotherapeutic target, we generated single‐cell RNA‐seq profiles for samples of one tumour from a patient with phase IIIC HGS OC and matched normal‐tumour pairs from a borderline OC patient. The samples were from patients who did not receive any chemotherapies or other treatments. For each sample, we took three different sections of the tissue for mixed sequencing (Figure 1A). After removing the batch effects and conducting regression analysis to account for the influence of the number of unique molecular identifiers (UMIs) and percentage of mitochondria‐derived UMI counts, we obtained transcriptomes of 16027 individual OC tumour‐derived cells and 7655 cells originating from the normal ovary for further analysis. Uniform Manifold Approximation and Projection (UMAP) visualization revealed the presence of nine distinct TME‐related clusters in OC samples (Figure 1B, Figure S1A and Table S1). The well‐established cell type markers used for cluster identification were acquired from the CellMarker database and previous studies.^12^, ^13^ We visualized proportional differences in specific marker gene expression among these main cell clusters using a bubble chart (Figure S1B), and the UMAP map displayed the expression of specific known markers supporting the accuracy of cluster identification (Figure 1C and Figure S1C). Next, the large‐scale copy number variations (CNVs) data showed that epithelial and fibroblast cells in HGSOC had higher CNV levels compared with other cell types and the same cell types in borderline tumours and normal ovaries (Figure 1D). Notably, no significant differences in CNV levels between normal and benign borderline tumours. These results confirmed cellular diversity and malignant cell types in OC TME.

Subsequently, we analyzed the proportion of various cell types to deep understanding of the prominent feature of OC TME. Unexpectedly, our data demonstrated that in the HGSOC samples, T cells accounted for 23.23%—second only to malignant epithelial cells 48.93%. The T cells represented a predominant cell type, although TILs are known to be limited in many tumour types. In borderline carcinoma TME, myeloid cells (32.41%) comprised the greatest proportion, while in normal ovary, epithelial cells (68%) comprised the greatest proportion (Figure 1E,F and Figure S1D). Gene Set Enrichment Analysis (GSEA) revealed that genes involved in the interferon response and antigen presentation pathways were enriched in T cells, thus supporting T cell prevalence played immunomodulatory role in tumour progression (Figure 1G). These data illustrated cellular diversity in different tumour types.

### 2.2 The preferentially enriched cell type among T cells in the OC TME are exhausted T cells

**FIGURE 2:**
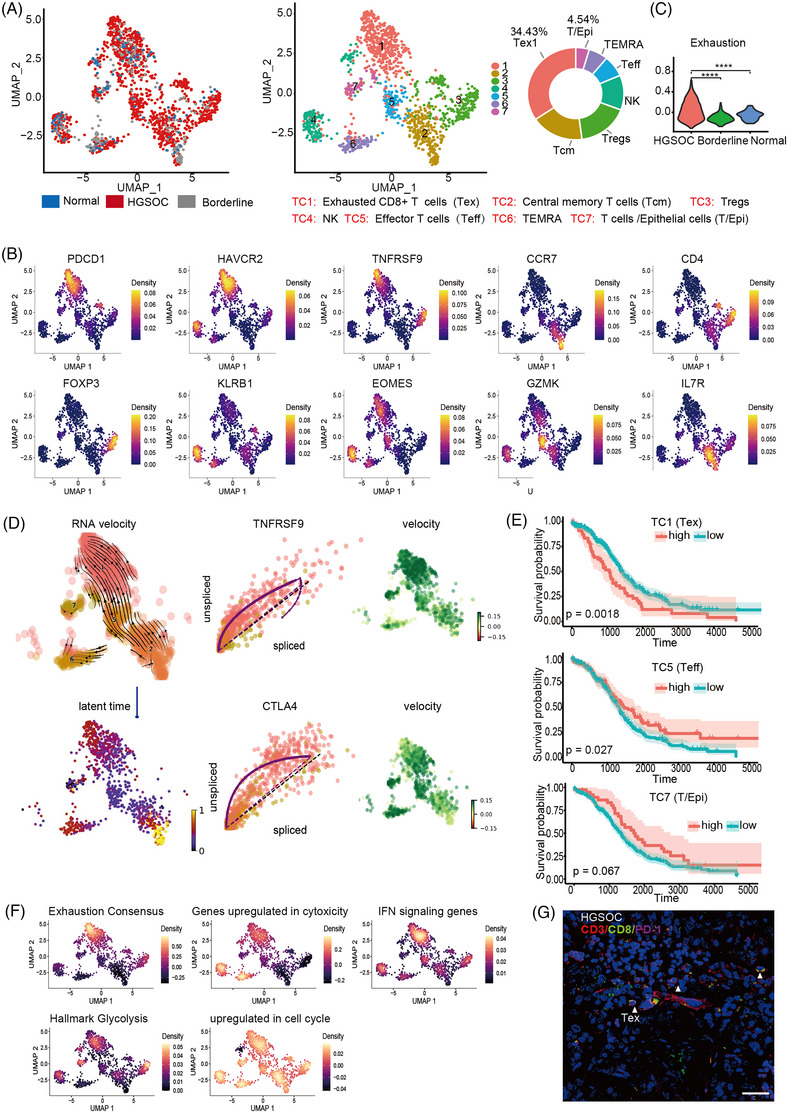
Exhausted infiltrated T cells are preferentially enriched in the ovarian cancer (OC) microenvironment. (A) Uniform Manifold Approximation and Projection (UMAP) plot of T cells, with each colour coded for seven major cell types and sample origin and Pie charts depicting the ratio of the two highest T cell types in OC tissues. (B) Expression levels of specific markers for each cell type are plotted on the UMAP. The colour key from purple to yellow indicated relative expression levels from low to high. (C) Violin plot demonstrating exhaustion score in different tissues. (D) RNA Velocity vectors projected onto the UMA indicating differentiation directionality of T cells. (E) Kaplan‐Meier curves for patients with different CD8^+^ T cell cluster scores based on cibersortx. (F) Significantly enriched Hallmark gene sets are plotted onto the UMAP. The colour key from purple to orange indicates relative expression levels from low to high. (G) Multicolour immunofluorescence staining showing the presence of exhausted CD3^+^CD8^+^ T cells, exemplified by high‐grade serous ovarian cancer (HGSOC) patient, labelled with white arrow. The scale bar represents 50 μm.

To explore the potential cell subtypes within the overall T cells of the OC samples, we re‐clustered the T cells into seven sub‐clusters (TC1–TC7), the TC1 subcluster was preferentially enriched and dominant among T cells (34.43%) (Figure 2A and Table S2). The top 10 significant differentially expressed genes (DEGs) for each cluster were visualized by heatmap (Figure S2A). The highly upregulated DEGs were enriched for genes involved in exhaustion, such as HAVCR2 (TIM‐3), LAG3, PDCD1 (PD‐1) and CTLA‐4, indicating that TC1 were exhausted T cells (Figure S2B). Further analysis showed that TC1 was in the final state of exhausted T cells (PD‐1^+^, TIM‐3^+^ LAG‐3^+^ CD69^+^CD39^+^ Tox ^+^Blimp^+^)^14^, ^15^ (Figure S2C). Then, we identified TC2 as central memory T cells (Tcm), TC3 as CD4^+^ regulatory T cells (Tregs), TC4 as NK, TC5 as effector CD8^+^ T (Teff), TC6 as TEMRA, TC7 as T/Epithelia cells (Figure 2B and Figure S2B). Exhaustion score data demonstrated the T cells of HGSOC had a significant exhaustion feature (Figure 2C).

To investigate the connections of TC1 and TC7, and to explore the possible origin of exhausted T cells in OC, we used RNA velocity analysis.^16^ This analysis showed an obvious path of T cell differentiation from TC2 to TC5 to TC7, eventually converging to TC1 (Figure 2D). In addition, transcriptional activation of inhibitory receptors (e.g., CTLA4 and TNFRSF9) in the gene expression dynamics resolved along latent time showed late differentiation by TC1 cells, which was recapitulated in pseudotime diffusion map analysis (Figure S2D). Taken together, we established the T cells differentiated from Tcm to Teff to Tex. Kaplan‐Meier curves for patients with different CD8^+^ T cell cluster scores based on cibersortx demonstrated that TC1 was significantly related to poor (*p* = .0018) in OC, while TC5 cells were correlated with good prognosis (*p* = .027), and TC7 were no significant correlation (*p* = .067) (Figure 2E).

Subsequently, we performed GSEA using the AddModuleScore function of Seurat to explore the possible functions of TC1.^13^ This analysis demonstrated the enrichment of specific genes associated with the exhausted state, supporting that the TC1 subcluster consisted of exhausted CD8^+^T cells (Figure 2F). Enrichment for IFN genes in TC1 suggested that persistent interferon signalling could be related to the exhaustion status of these CD8^+^T cells. The tumours evade the host immune response through disruption of T cell metabolism,^17^, ^18^, ^19^ which was reflected by enrichment for glycolysis‐related genes in TC1. Furthermore, our data agree with other studies that reported high expression of proliferation‐related genes in inhibitory receptor‐expressing T cells (Figure 2F). The Kyoto Encyclopedia of Genes and Genomes pathway analysis showed the 10 most significantly enriched signalling pathways in TC1 (Figure S2E). We used SCENIC analysis to explore the key transcription factors (TFs) that cause OC‐specific T‐cell exhaustion. The data suggested that the participation of previously unrecognized candidate TFs in OC‐specific T cell exhaustion, including IRF2 (immunosuppression), CEBPB (lipid accumulation), and KDM5A (potential oncogene associated with tumorigenesis and metastasis^20^), were upregulated in the TC1 subcluster (Figure S2F). In sum, our results support that the TC1 subcluster recapitulated exhausted cell characteristics.

The immunofluorescence staining for PD‐1 in CD8^+^ lymphocytes confirmed the presence of exhausted T cells in clear cell carcinoma, HGSOC, and endometrial carcinoma (Figure 2G and Figure S2G). These results showed that exhausted CD8^+^T cells are present in a variety of OCs. Collectively, TILs, in particular, exhausted T cells, represented an abundant subpopulation of immune cells in the TME of OC and implied that these exhausted T cells could potentially perform a range of functions in the development of cancer.

### 2.3 Identification genes uniquely associated with OC‐exhausted CD8^+^ T cells

**FIGURE 3:**
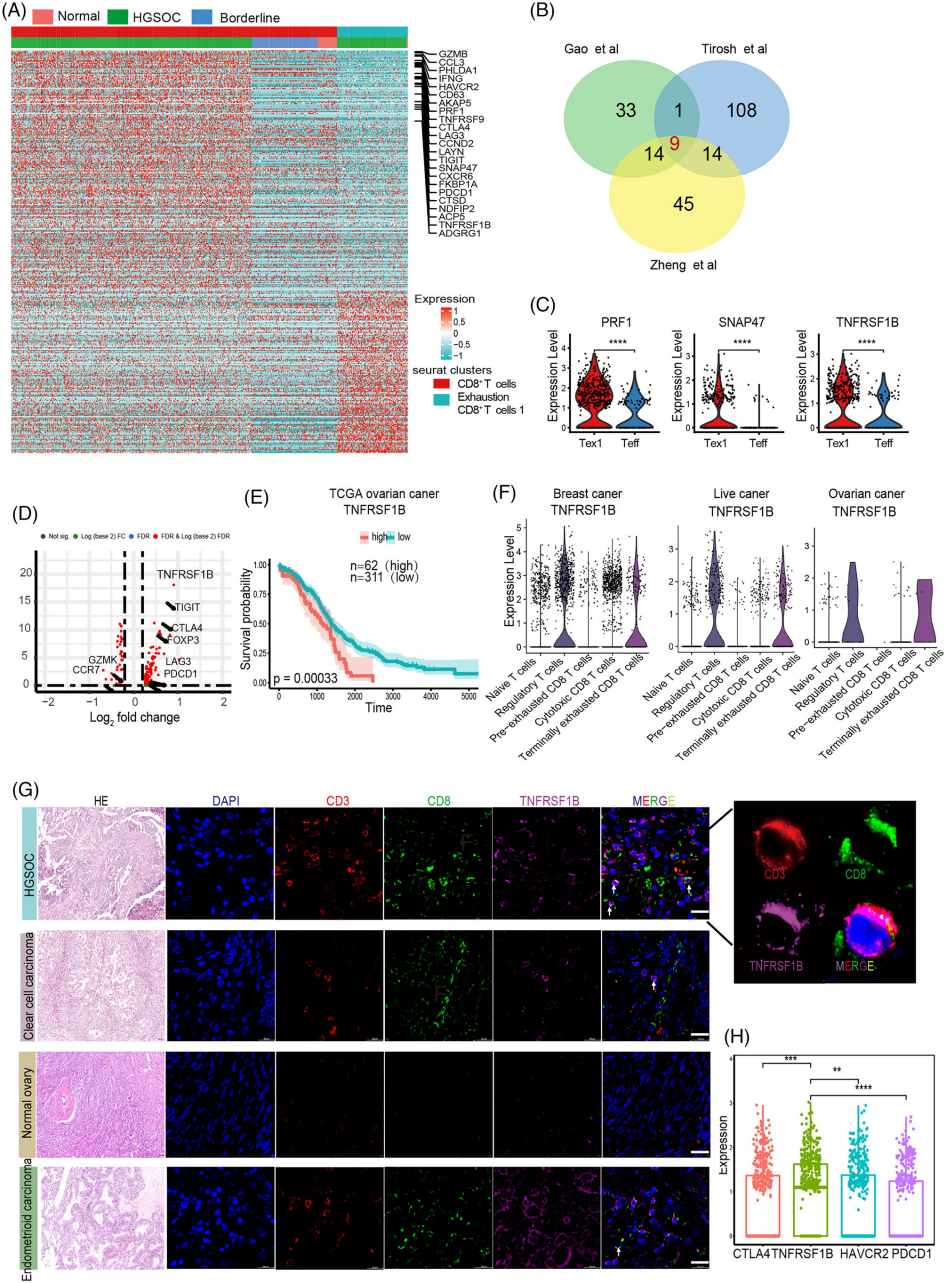
A series of markers specifically associated with ovarian cancer (OC)‐infiltrating exhausted CD8^+^ T cells. (A) Heatmap demonstrating differentially expressed genes between exhausted and non‐exhausted tumour‐infiltrating lymphocytes (TILs). The top bar shows the exhaustion states and the lower bar indicates tissue origins. The top differentially expressed gene (DEG) and known exhausted maker were denoted to the right. (B) The Venn graph shows the overlap of exhausted CD8^+^T cell genes identified in this study with those from previous studies by Zheng et al. and Tirosh et al., nine overlapped genes were recognized. (C) Violin plot demonstrating gene expression levels in exhausted CD8^+^T cells (Tex1) and non‐exhausted CD8^+^T cells (Teff). ****p < .0001; Student's t‐test. (D) Volcano plots showing significantly differentiated genes according to TNFRSF1B high and low expression in T cells from OC samples. (E) Kaplan‐Meier curves showing patients with higher expression of TNFRSF1B in tumors had poor prognosis based on TCGA OC data. (F) Violin plot showing the expression of TNFRSF1B in various T cell subtypes of the previous scRNA‐seq. (G) Multicolor immunofluorescence staining showing the presence of CD3^+^CD8^+^ TNFRSF1B^+^ T cells in various ovarian cancer subtypes and normal ovary, using CD3, CD8 and TNFRSF1B antibodies. The scale bar represents 20 μm. (H) Boxplots of exhausted marker gene expression on T cells.

To identify genes specifically associated with exhausted CD8^+^ T cells in OC, we compared the transcriptomes of exhausted T cells (Tex) and non‐exhausted T cells (Teff), generated a list of 57 special exhausted genes (adjusted p‐value < .05, fold change R ≥ .25) (Figure 3A and Figure S3A and Table S3). We obtained a consensus list of exhausted genes that overlapped with two previous studies which reported exhaustion features of T cells in melanoma and liver cancer.^21^, ^22^ A total of nine genes with high expression on Tex1 cells compared with that on Teff cells overlapped among the three studies (Figure 3B). Although six genes were well‐known exhaustion markers in the list, three genes (TNFRSF1B, SNAP47 and PRF1) have not been thoroughly linked to T cell exhaustion (Figure 3C and Figure S3B). Differential expression analysis revealed that TNFRSF1B ‐high T cells expressed a few immune‐inhibitory factors, such as CTLA4, PD‐1, TIGIT and CCL4L2, indicating a dysfunctional and exhausted phenotype (Figure 3D). By interrogating The Cancer Genome Atlas (TCGA) OC survival data, we found that elevated expression of TNFRSF1B was related to poor prognosis (TNFRSF1B *p* = .00033, log‐rank test) after normalizing the effects of infiltrated T cell levels through CD3 expression (Figure 3E). We examined other bulk RNAseq databases^23^ and confirmed that high TNFRSF1B expression was associated with poor prognosis (*p* = .031, Figure S3C). Taken together, TNFRSF1B was identified as a potential exhausted CD8^+^ T cell marker, and high expression of TNFRSF1B was related to poor prognosis.

We then detected the expression of TNFRSF1B on exhausted T cells in published single‐cell RNA sequencing data from breast cancer, intrahepatic cholangiocarcinoma, and other OC studies. These analyses showed that TNFRSF1B was highly expressed on terminally exhausted CD8^+^ T cells and Tregs, and indicated that TNFRSF1B could be a T cell exhaustion marker (Figure 3F). To clarify the presence of CD8^+^TNFRSF1B^+^ cluster in OC patients, immunofluorescence staining was performed. We detected a clear TNFRSF1B signal on CD8^+^ T cells on tumour sections from HGSOC, clear cell carcinoma, and endometrial carcinoma patients, but not in normal ovary tissue (Figure 3G). Additionally, the expression of TNFRSF1B was much higher than the other exhausted markers, indicating that TNFRSF1B was a potentially reliable target of immune therapy for OC (Figure 3H). These results provided evidence that TNFRSF1B could affect and regulate exhausted T cells, and is a new candidate exhausted marker for OC.

### 2.4 TNFRSF1B is induced in activated CD8^+^ T cells and suppresses IFN‐γ production

**FIGURE 4:**
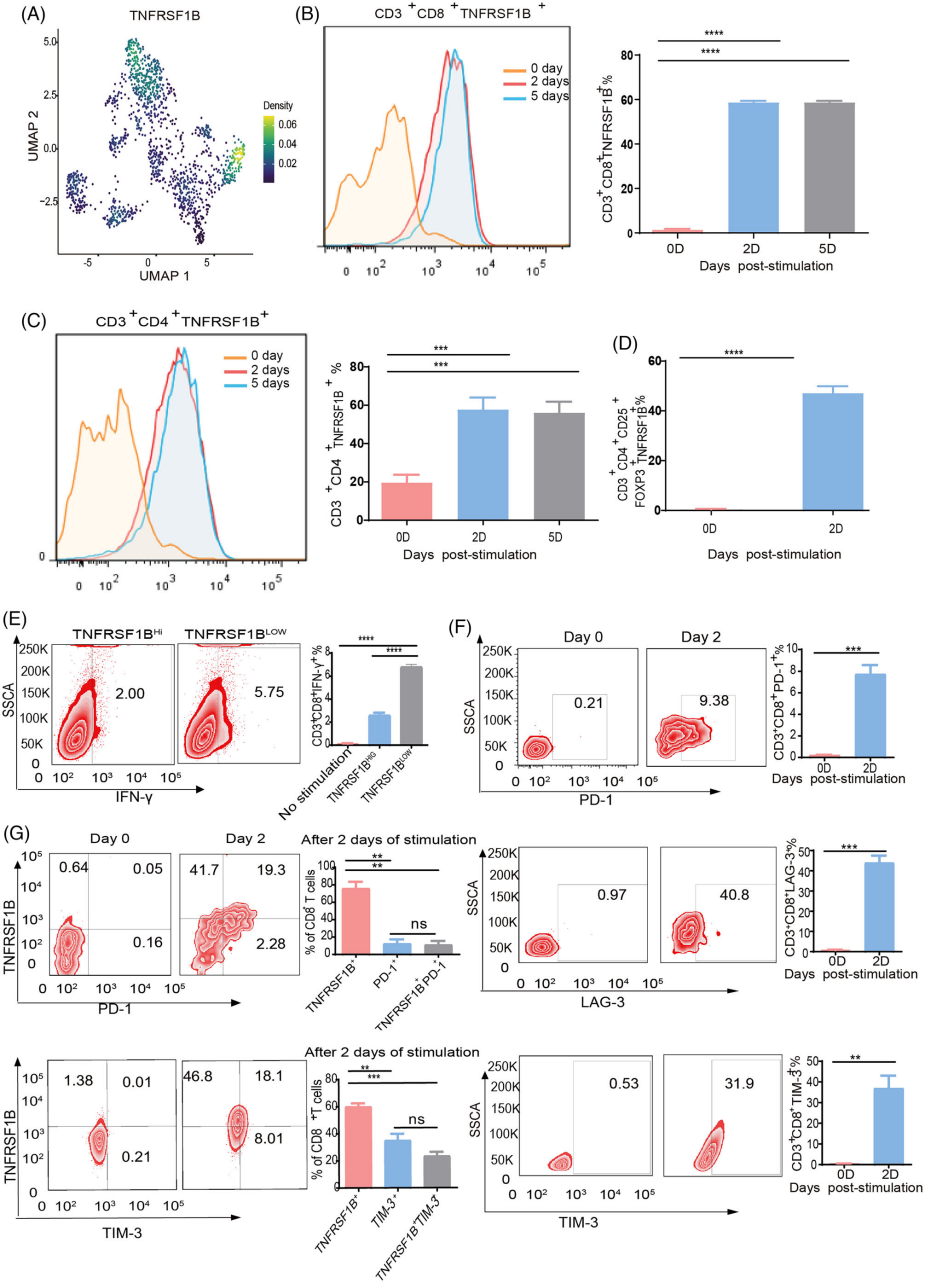
TNFRSF1B is induced in activated CD8+ T cells and suppresses interferon (IFN)‐γ production. (A) Expression levels of TNFRSF1B for each cell type are plotted onto the Uniform Manifold Approximation and Projection (UMAP). The colour key from blue to yellow indicated relative expression levels from low to high. (B–D) Human peripheral blood mononuclear cells (PBMCs) were stimulated in vitro with anti‐CD3 and anti‐CD28 monoclonal antibodies for 2 and 5 days. Gated on each indicated population (CD8 T cells: CD3^+^CD8^+^, CD4 T cells: CD3^+^CD4^+^, Tregs: CD3^+^CD4^+^CD25^+^Foxp3^+^), TNFRSF1B expression was determined by Flow cytometry (left), and statistical analysis (right). Data represents mean ± SEM n = 6, ****p < .0001. (E) Representative flow cytometry analysis (left) and bar graph (right) of human IFN‐γ in the cell supernatant of sorted TNFRSF1B^high^ and TNFRSF1B^low^ CD8^+^T cells. ****p < .0001. Data represents mean ± SEM *n* = 3. (F) Human PBMCs were similarly stimulated as in Figure 4B for 2 days. Gated on CD8^+^T cells, TNFRSF1B, PD‐1 and LAG‐3 expressions were determined by flow cytometry (FACS). Data represents mean ± SEM *n* = 6. (G) Flow cytometry plots showing TNFRSF1B and exhausted maker expression on CD8^+^T cells after stimulated in vitro with anti‐CD3 and anti‐CD28 monoclonal antibodies for 2 days.

Because no association between TNFRSF1B and tumour‐infiltrating exhausted CD8^+^ T cells has been previously reported in HGSOC, we further characterized its expression and regulation in blood‐isolated CD8^+^T cells in vitro. Our scRNA‐seq data revealed a high expression pattern of TNFRSF1B in both tumour Tregs and exhausted T cells (Figure 4A). We then verified the expression of both CD8^+^ T cells and Tregs isolated from human peripheral blood mononuclear cells (PBMCs) by flow cytometry (FACS). In the resting stage, TNFRSF1B was not expressed on either CD4^+^ T or CD8^+^ T cells (Figure S4A). However, after T cell activation with anti‐CD3 and anti‐CD28 antibodies for two days, TNFRSF1B could be clearly detected on > 50% CD8^+^ and CD4^+^ T cells, > 40% Tregs, and could still be detected five days post activation (Figure 4B–D). Next, we divided TNFRSF1B positive cells into high‐ and low‐expression cells through FACS. Interestingly, the CD8^+^TNFRSF1B^high^ T cells produced significantly less IFN‐γ than CD8^+^TNFRSF1B^low^ T cells (Figure 4E), supporting the suppressed role of TNFRSF1B in CD8^+^ T cells. Then, we analyzed the prognosis and expression of other candidate genes, including PRF1 and SNAP47, which could be induced in CD4^+^ and CD8^+^ T cells upon activation, but no significant differences in IFN‐γ production (Figure S4B–D). These findings therefore corroborated transcriptomics data showing that TNFRSF1B expression could be induced on both Tregs and CD8^+^ T cells, and high TNFRSF1B expression on CD8^+^ T cells associated with lower IFN‐γ.

At present, blockade of inhibitory molecules such as the PD‐1/PD‐L1 is the most widely used in immunotherapy, but unfortunately, the OC response to PD‐L1/PD‐1 blockade is weaker than that of other tumour types. In our study, the classical inhibitory molecules PD‐1, LAG‐3, and TIM‐3 were also induced on active CD8^+^ T cells. 9% of active CD8^+^ T cells highly expressed PD‐1, 40% expressed LAG‐3 and 31% expressed LAG‐3, while 59% expressed TNFRSF1B (Figures 4B,F), and still could be detected five days post activation (Figure S4E). Moreover, TNFRSF1B could be detected on majority CD8^+^PD‐1^+^ T cells, and no significant difference in cell number of TIM‐3^+^ T cells and TNFRSF1B^+^TIM‐3^+^ T cells, LAG‐3^+^ T cells and TNFRSF1B ^+^LAG‐3^+^ T cells, suggesting TNFRSF1B^+^T cell type could cover all these three exhausted cell types (Figures 4G,F). Correlation analysis indicated that TNFRSF1B expression was strongly correlated with both PD‐1 (*p* = 1.23e‐22) and LAG‐3 (*p* = 1.43e‐05) (Figure S4G). The expression pattern of TNFRSF1B suggested the preferential and clonal enrichment of CD8^+^TNFRSF1B^+^ T cells in the OC microenvironment. In sum, these results indicated the possible value of TNFRSF1B as a marker for T cell exhaustion in OC.

### 2.5 TNFRSF1B expression is closely associated with OC clinical malignancy and is a poor prognosis marker

**FIGURE 5:**
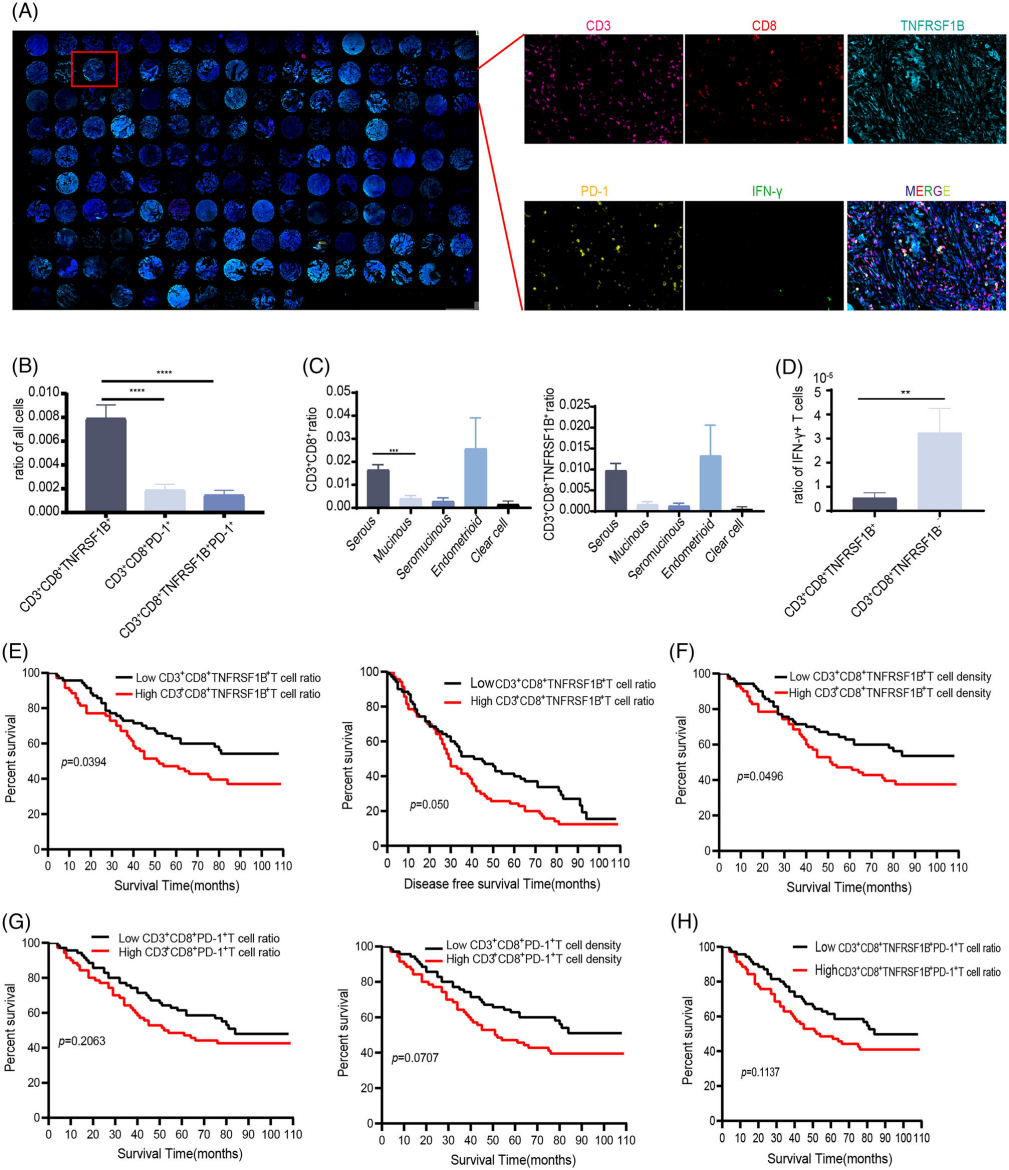
TNFRSF1B expression is closely associated with ovarian cancer (OC) clinical malignancy and is a poor prognosis marker (A) Multicolor immunofluorescence staining of ovarian TMA for CD3, CD8, TNFRSF1B, programmed cell death 1 (PD‐1) and interferon (IFN)‐γ. (B) Bar plots showing the ratio of CD3^+^CD8^+^PD‐1^+^, CD3^+^CD8^+^TNFRSF1B^+^T and CD3^+^CD8^+^ TNFRSF1B^+^ PD‐1^+^ T cells relative to the total cells. (C) Bar plots showing the ratio of CD3^+^CD8^+^ T (left) and CD3^+^CD8^+^TNFRSF1B^+^ T cells (right) in different OC subtypes. (D) Bar plots showing the ratio of IFN‐γ^+^ T cells in CD3^+^CD8^+^TNFRSF1B^+^ and CD3^+^CD8^+^TNFRSF1B^−^ T cells group. (E) Ovarian cancers with lower levels of TNFRSF1B ratio compared to tumours with higher TNFRSF1B ratio were associated with good survival (left) and disease‐free survival (right). (F) Ovarian cancers with lower levels of TNFRSF1B density compared to tumours with higher TNFRSF1B density were associated with good survival. (G) Overall survival for OC patients according to PD‐1 ratio (left) and density (right). (H) Overall survival for OC patients according to CD3^+^CD8^+^TNFRSF1B^+^PD‐1^+^ ratio, Log‐rank *p*‐values are shown.

To determine the potential role of infiltrated CD3^+^CD8^+^TNFRSF1B^+^T cells in clinical progression, we immunostained tumour sections from 140 OC patients with 9 years follow‐up to detect CD3, CD8, TNFRSF1B, PD‐1, and IFN‐γ expression. The quantification of the digital slides revealed higher proportions of CD3^+^CD8^+^TNFRSF1B^+^T cells than CD3^+^CD8^+^PD‐1^+^T cells (Figure 5A,B). For different OC tissue types, the serous tumours had more CD3^+^CD8^+^ T cells and CD3^+^CD8^+^TNFRSF1B^+^T cells ratio compared to mucinous, and the endometrioid tumour had the highest ratio in all subtypes (Figure 5C). We also examined the IFN‐γ expression, the data demonstrated that the IFN‐γ level in the CD3^+^CD8^+^TNFRSF1B^+^ T cell group was significantly lower in the CD3^+^CD8^+^TNFRSF1B^−^ T cells group (Figure 5D). In the case of CD3^+^CD8^+^TNFRSF1B^+^T cells, a high ratio correlated with malignant histologic subtype (*p* < .0001) (Table 1). Furthermore, higher CD3^+^CD8^+^TNFRSF1B^+^ ratio and density both correlated with worse overall survival (*p* = .0394, *p* = .0496) (Figure 5E,F), while there was no significant difference in overall survival between high and low CD3^+^CD8^+^PD‐1^+^T cells ratio and density (Figure 5G). We did not observe significant difference in overall survival between high and low CD3^+^CD8^+^TNFRSF1B^+^PD‐1^+^T cells ratio (Figure 5H), indicating that CD3^+^CD8^+^TNFRSF1B^+^T cells ratio can reflect the survival of ovarian patients without combined PD‐1 expression. Additionally, we found CD3^+^CD8^+^TNFRSF1B^+^T cells ratio correlated with PD‐L1 and Ki67 expression (Table 2), suggesting that CD3^+^CD8^+^TNFRSF1B^+^T cells ratio can replace multiple indicators to reflect clinical malignant character.

**TABLE 1:** Clinicopathological features in ovarian cancer (OC) patients and the correlation with CD3^+^CD8^+^TNFRSF1B^+^ subpopulation ratio.

Clinicopathological features	Total no. (%) (*N* = 140)	CD3^+^CD8^+^TNFRSF1B^+^ subpopulation ratio		
		Low (*n* = 70)	High (*n* = 70)	*p*‐Value
**Age**				.6118
≤50	67 (47.86)	35 (50.00)	32 (45.71)	
>50	73 (52.14)	35 (50.00)	38 (54.29)	
**Pathological stage**				.0929
Early stage (I‐II)	19 (13.57)	9 (12.86)	10 (14.29)	
Advanced stage (III)	95 (67.86)	43 (61.43)	52 (74.29)	
Unknown	26 (18.57)	18 (25.71)	8 (11.43)	
**Histologic subtype**				**<.0001** ^a^
Serous	74 (52.86)	27 (38.57)	47 (67.14)	
Mucinous	30 (21.43)	25 (35.71)	5 (7.14)	
Endometrioid	15 (10.71)	4 (5.71)	11 (15.71)	
Seromucinous	4 (2.86)	3 (4.29)	1 (1.43)	
Others	17 (12.14)	11 (15.71)	6 (8.57)	
**Clinical stage**				.0723
1	8 (5.71)	7 (10.00)	1 (1.43)	
2	34 (24.29)	16 (22.86)	18 (25.71)	
3	68 (48.57)	36 (51.43)	32 (45.71)	
4	30 (21.43)	11 (15.71)	19 (27.14)	
**Distant metastasis**	30 (21.43)	11 (15.71)	19 (27.14)	.0994
**Relapse**	113 (80.71)	52 (74.29)	61 (87.14)	.0539

^a^ statistically significant.

**TABLE 2:** Correlation between CD3^+^CD8^+^TNFRSF1B^+^ subpopulation and KI67 and PD‐L1.

	CD3^+^CD8^+^TNFRSF1B^+^ subpopulation ratio			
Biomarker	Low (*n* = 70)	High (*n* = 70)	*p*‐Value^a^	R^b^
**Ki67**			**.0002** ^c^	.29926
Low	52 (74.28)	30 (42.86)		
High	18 (25.72)	40 (57.14)		
**PD‐L1**			**<.0001** ^c^	.40422
Low	54 (77.14)	31 (44.29)		
High	16 (22.86)	39 (55.71)		

^a^ Chi‐squared Test.

^b^ Spearman correlation coefficient.

^c^ Statistically significant.

### 2.6 Blockade of TNFRSF1B inhibits tumour growth through profoundly remodeling the immune microenvironment in OC mouse model

**FIGURE 6:**
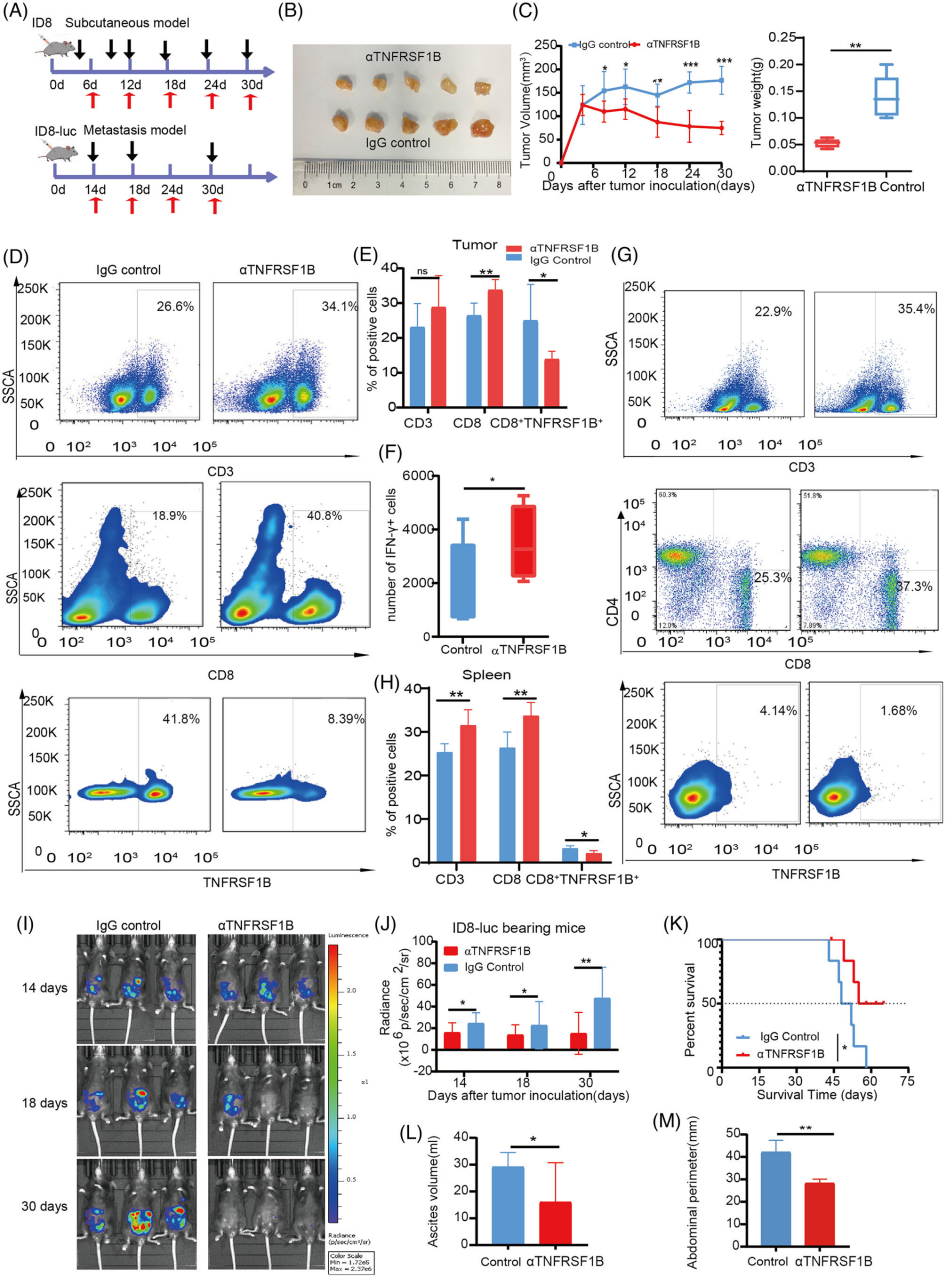
Blockade of TNFRSF1B inhibits tumour growth in the ovarian cancer mouse model. (A) Workflow showing the experimental process of the subcutaneous and metastatic mice study. Black arrow: tumour size measurement, red arrow: dosing time. (B) Tumours were isolated and photographed from subcutaneous ID8‐bearing mice after being treated with 200 mg anti‐TNFRSF1B or isotype‐matched control antibody IgG via intraperitoneal injection (*n* = 5). (C) Tumor growth curves (left) and tumour weight (right) at the endpoint in subcutaneous tumours at day 30 were measured (*n* = 5). (D) and (E) Representative flow cytometry analysis and quantification of CD3^+^T, CD8^+^T and CD8^+^TNFRSF1B^+^T cells in tumour infiltrating lymphocytes (TILs) in subcutaneous tumours at day 30 (*n* = 5). (F) Quantification of flow cytometry results of CD8^+^IFN‐γ^+^ T cells in subcutaneous tumors from mice receiving the indicated treatments as described in (B). (G, H) Representative flow cytometry analysis and quantification of CD3^+^T, CD8^+^T and CD8^+^ TNFRSF1B^+^T cells in splenic‐T cells in subcutaneous tumours at day 30 (n = 5). (I) ID8‐Luciferase cells were implanted intraperitoneally in C57BL/6 mice. Mice were treated with 200 mg anti‐TNFRSF1B or isotype‐matched control antibody IgG via intraperitoneal injection as indicated according to the initial bioluminescence (*n* = 5). (J) Quantification of bioluminescence. Data are presented as means ± SEM of n mice, as indicated on each panel. Statistical significance was determined by two‐way ANOVA. *p < .05 and **p < .01. (K) Kaplan–Meier survival analysis of metastatic ID8‐luc tumors treated as in I. (L) and (M) Representative ascites volume and abdominal perimeter of metastatic mice at day 30 (*n* = 5). Statistical significance was calculated by unpaired two‐tailed Student's t‐tests. *p < .05, **p < .01 and ***p < .001.

To determine the antitumor effect of TNFRSF1B in vivo, we used a syngeneic subcutaneous tumour model and a metastatic OC model was used. For the subcutaneous tumour model, IgG isotype control antibody and anti‐TNFRSF1B antibody was injected intraperitoneally every 6 days, and the tumour volume was measured on day 5, 9, 12, 18, 24 and 30 after ID8 injection (Figure 6A). Mice were euthanized on day 30, and tumours were isolated, weighed, photographed, and FACS analyzed. TNFRSF1B antibody caused a pronounced suppression effect on tumour growth after five treatments, lower tumour volume and tumour weight at the endpoint were observed in the subcutaneous tumour model (Figure 6B,C). Next, we evaluated how anti‐TNFRSF1B antibody treatment affected T‐cell responses and remodelling of the immune microenvironment. The immune cell infiltration in the TME of the subcutaneous tumour model was analyzed, and we found that TNFRSF1B blockade did not affect the infiltration of CD3^+^T cells but affected CD8^+^T cells (Figure 6D,E and Figure S5A). Furthermore, the anti‐TNFRSF1B antibody treatment increased the proportion of CD8^+^IFN‐γ^+^ T cells compared to IgG control, indicating that the cytotoxic function of CD8^+^ T cells was activated (Figure 6F). In splenic isolated T cells, we observed the same trend (Figure 6G,H and Figure S5B). In addition, decreased abundance of TNFRSF1B in CD8^+^ T cells in the tumour and spleen, indicating that anti‐TNFRSF1B antibody treatment could reduce the suppressed CD8^+^TNFRSF1B^+^ T cells (Figure 6E,H). These results demonstrated that the anti‐TNFRSF1B antibody could alleviate the tumour burden by affecting T cell responses and reversing immunosuppression in ID8 tumour‐bearing mice.

For the metastatic OC model, the same antibody treatment was performed on days 14, 18, 24 and 30, and tumour fluorescence was measured on days 14, 18 and 30 after injecting ID8‐luc (Figure 6A). The in vivo imaging of tumours from mice with intraperitoneal injection demonstrated 100% metastatic tumour growth in control mice but 20% (1/5) in mice after being treated with anti‐TNFRSF1B antibodies (Figure 6I,J and Figure S5C,D). TNFRSF1B blockade could inhibit ID8‐luc cell metastasis, with a 50% survival rate while 0% survival in the control group after 60 days of treatment (Figure 6K). Ascite volume and abdominal perimeter in ID8‐bearing mice were significantly reduced after anti‐TNFRSF1B antibody treatment compared to the IgG control group (Figure 6L,M and Figure S5E). These results demonstrated the key role of TNFRSF1B in inhibiting tumour growth in OC.

## 3 DISCUSSION

OC is a highly aggressive and the most lethal gynecologic malignancy with less than 20% 5‐year survival. Therefore, more effective therapy alternatives for OC are urgently needed. While cancer immunotherapy is effective for patients with a specific set of indications, a relatively large subset of OC patients still exhibits poor overall survival and weak immunotherapy response. The specific factors underlying this attenuated treatment response have remained unclear, mostly due to unpredictable functional or phenotypic heterogeneity in the immunosuppressive TME.^24^

Now, increasing bulk RNA‐seq and scRNA‐seq analyses were used to characterize the diversity of cell populations. To build on the findings of other studies that explored the TME of OC tumours,^25^, ^26^ and further identify previously unrecognized therapeutic targets for OC patients, we establish high‐resolution landscapes of the TME in different levels of malignant OC. Our results show that, overall, TILs are almost exclusively present in HGSOC tumour tissues, whereas myeloid cells are mainly found in borderline tissues, and normal epithelial cells predominate normal ovary tissues.

In our study, although HGSOC tumours harbour a significant level of TILs, the tumour is still in malignant progression, those TILs are inferred to be immunosuppressive or dysregulated. To expound on the status and characteristics of these TILs, we focused on characterizing T cell subpopulations. We confirmed that TILs were dominantly comprised of exhausted T cells. By interrogating the exhausted T cell subcluster, we identified a set of novel and highly upregulated candidate markers associated with T cell exhaustion, such as TNFRSF1B which might reveal the T cell exhaustion signature and associate with T cell function. It was reported that TNFRSF1B was highly expressed in suppressor immune cells, such as Tregs and myeloid inhibitory cells, NK cells, and other cell types.^27^, ^28^ The responses of these cells to TNFRSF1B signalling are different and even result in the opposite effect. Because of the complicated mechanism of TNFRSF1B, how to regulate the biological function of these cell types in TME remains elusive, while its expression and function on exhausted T cells have remained poorly understood.

Our analyses showed that TNFRSF1B is preferentially expressed on exhausted T cells, and in vitro, assays first verified its induction following activation of both CD8^+^ T cells and Tregs. Furthermore, we confirmed that high TNFRSF1B expression in primary CD8^+^T cells resulted in reduced IFN‐γ production, which is necessary for tumour‐killing activity in T cells, determine the mechanistic factors leading to the pro‐tumorigenic effects of TNFRSF1B. Additionally, recent scRNA‐seq and functional T‐cell studies have shown that exhausted T cells are highly proliferative,^29^, ^30^ and since that TNFRSF1B activation can initiate pro‐survival response pathways in these cells via nuclear factor kappa B (NF‐kB)‐dependent signalling,^31^, ^32^ suggesting that this gene could drive the maintenance of exhausted T cell populations in the TME. Although these results strongly support TNFRSF1B as a negative regulator of IFN‐γ, further mechanistic studies are necessary. Additionally, our results suggested that low PD‐1 and CTLA‐4 expression on exhausted T cells in OC may cause the poor effectiveness of antibody therapy (Figure S6), while TNFRSF1B was highly expressed and could suppress the function of CD8^+^T cells. Other reports have also shown that the overall abundance of TNFRSF1B gene expression is markedly higher than that of CTLA‐4 and PD‐L in OC tissues.^33^ Additionally, it was reported that TNFRSF1B antibody could elicit anti‐tumour activity and improve the effect of PD‐1 in syngeneic mice tumour models.^34^ These results showed that TNFRSF1B was a potential target of immunotherapy for OC. Collectively, these findings can guide further study of TIL characteristics and inform the development of effective immunotherapy strategies. Our study thus broadens our understanding of factors contributing to immunotherapy failure in HGSOC, and points to the clinical value of TNFRSF1B, in particular, as a marker and potential immunotherapy target of OC.

In conclusion, our single‐cell transcriptome analysis has been applied to TME components from OC patients revealing the cellular diversity of the TME of HGSOC, borderline OC, and normal ovary. Further characterization analysis reveals the T cell exhaustion signature, one of the genes, TNFRSF1B, is upregulated on activated CD8^+^ T cells and Tregs repress the CD8^+^ T cell functions in PBMC. The analysis of OC samples from 140 patients provided significant insights that TNFRSF1B expression is closely associated with OC clinical malignancy and is a poor prognostic marker. Blockade of TNFRSF1B inhibits tumour growth by profoundly remodelling the immune microenvironment in OC mouse models. It was reported that TNFRSF1B is highly expressed in Tregs and TNFRSF1B signalling results in augmented Tregs proliferation through NF‐kB signalling. Blocking TNFRSF1B could directly inhibit tumour growth by suppressing Treg function. Therefore, the good prognosis and anti‐tumour functions caused by blocking TNFRSF1B are the result of a variety of cells. Token together, TNFRSF1B would be an immune system marker of suppressive T cell types, and be an attractive drug target for its multiple functions.

## 4 EXPERIMENTAL SECTION

### 4.1 Patient samples

OC samples were obtained at Beijing Obstetrics and Gynecology Hospital, three tissue samples were used for scRNA‐seq and six samples for immunofluorescence. All patient samples were included after signing informed consent. Furthermore, five OC patients and three normal healthy donors for obtaining PBMC were included in this study. The study was carried out according to the Declaration of *Beijing Obstetrics and Gynecology Hospital* and approved by the local medical ethical committee, and the approval number was 2018‐KY‐048‐01.

### 4.2 Tissue dissociation and single cell preparation

We collected fresh tissues in MACS Tissue Storage Solution(130‐100‐008F)on ice to keep cell viability. Then, we employed a tumour dissociation kit (MACS, 130‐095‐929) to generate single‐cell suspensions, and dissociated at 37°C for 6 min with gentle shaking. Note that, a 40 μm nylon cell strainer was used to filter the cell suspensions (Falcon, 352340). We removed the red blood cells and used AO/PI fluorescent dyes (Logos Biosystems, F23001) to stain single cells. Finally, LUNA (Logos Biosystems, LUNA‐STEM) was used to detect cell viability.

### 4.3 Peripheral blood mononuclear cells

We collected the venous blood (10 mL) in sodium‐heparin collection tubes and used Ficoll density gradient centrifugation to isolate PBMCs. Finally, the PBMCs were cultured in a complete 1640 medium with 10% fetal bovine serum (FBS; Gibco) and antibiotics.

### 4.4 T cell activation and FACS analysis

PBMCs were cultured in the complete 1640 medium with 10% FBS and antibiotics. At 2‐ and 5‐days post‐stimulation with CD3/CD28, cells were washed and incubated with antibodies (CD3 (BioLegend), CD8 (BioLegend), CD4 (BioLegend), CD25 (BioLegend), TNFRSF1B (BioLegend), FOXP3 (BioLegend), PD‐1 (BioLegend), LAG‐3 (BioLegend), TIM‐3 (BioLegend). For intracellular staining, we permeabilized cells using the Fixation and Permeabilization Kit^14^ to permeabilize the cells, then stained with IFN‐γ antibodies. The samples were detected by FACS and the data were processed using FlowJo software7.6. The antibodies and reagents are listed in Table S5.

### 4.5 Immunofluorescence

The paraffin sections were fixed in 4% paraformaldehyde (PFA) for 15 min to preserve the tissue structure, Subsequently, the sections were permeabilized using 0.1% Triton for an additional 15 min to allow antibody penetration. After the samples were blocked with goat serum, the samples were incubated with the primary antibody for 12 h at 4°C, then stained by the secondary antibodies for 2 h at 37°C in the dark. Finally, the sections were stained with 4′,6‐diamidino‐2‐phenylindole for 15 min to visualize the cell nuclei. Prepared sections were imaged using the Nikon A1 confocal microscope.

### 4.6 Subcutaneous OC model

The experimental animals were maintained in compliance with Animal Research Center guidelines and the Animal Care and Use Committee of Tsinghua University. The mice were housed in a pathogen‐free environment and according to the guidelines of the International Association for Assessment. We purchased female C57BL/6 mice, six‐week‐old, from Vital River (China). A total of 10^7^ ID8‐luc cells were subcutaneously injected into mice. Tumour‐bearing mice were randomly divided into two groups when the tumour size was 100 mm3 (1/2ab,^2^ where a was long diameter and b was short diameter of tumours). IgG isotype control antibody and anti‐TNFRSF1B antibody (200 μg/mouse) were intraperitoneally injected every 6 days. Mice were euthanized on day 30, and tumours were isolated, weighed, photographed, and FACS analyzed.

### 4.7 Metastatic OC model

For the metastatic prostate cancer model, C57BL/6 mice were intraperitoneal injected with 5 × 10^6^ ID8‐luc cells. The IVIS Imaging System was used to monitor the growth and metastasis of the tumour. We divided the tumour‐bearing mice into two groups randomly. IgG isotype control and anti‐TNFRSF1B (200 μg per dose) were administered intraperitoneally. The survival of mice was monitored and recorded.

### 4.8 Tumour infiltration cell isolation and FACS analysis

We minced the tumours which dissociated from treated mice, then digested them to single cell suspensions with the Tumour Dissociation Kit. Spleens were ground and filtrated through 70 μm filters to generate single cells, which were stained with antibodies for 20 min. For intracellular staining, we used the Fixation and Permeabilization Kit^14^ to further permeabilize, then stained the cells with appropriate antibodies. Finally, we used FACS to detect the cell samples and FlowJo software 7.6 to process the data.

### 4.9 Statistical analyses of TMA data

In these analyses, CD3^+^CD8^+^TNFRSF1B^+^T cells, CD3^+^CD8^+^PD‐1^+^T cells ratio and density were divided into low and high categories. These cutoff values were determined by using the median. We used the Kaplan–Meier survival curve with the Log‐Rank test to perform survival analysis. Spearman correlation tests were performed to determine if CD3^+^CD8^+^TNFRSF1B^+^T cells ratio correlated with other tumour markers. A level of 5% was used to determine significant statistical significance. We used SAS Version 9.4 to conduct the analyses.

### 4.10 Single‐cell RNA sequence data pre‐processing

The single cells were loaded in accordance with the instruction of the kit for Chromium single cell 3′ reagent. Library preparation was performed according to the 10x Genomics Chromium platform guidelines, and the resulting libraries were sequenced using the Illumina NovaSeq 6000 System. The low‐quality cells (less than 300 genes per cell, less than 3 cells per gene and more than 20% mitochondrial genes) were processed to filter out. The passing reads were processed through the Cell Ranger 3.0.1. We aligned the raw sequence files to the human GRCh38 reference genome using the STAR algorithm.^35^ Next, the gene‐barcode matrix, which contained gene expression counts and barcoded cells, was generated and then processed with Seurat v3.^36^ Finally, the data of UMI were normalized by log‐transformed. The highly variable genes (HGVs) were used to incorporate samples into the amalgamated dataset, and the amalgamated cells‐by‐genes matrix was scaled by dividing the centred expression by the standard deviation.

### 4.11 Dimension reduction and unsupervised clustering

The Seurat package and CellMarker database were used to analyze the cell types. HGVs were applied to perform unsupervised clustering of the PCA. PCs 1–30 were used to identify different cell populations. For sub‐clustering, were searched for variable genes first, then reduced dimensionality, and finally clustered to a limited dataset, variable genes were first found, then dimension was reduced, and finally clustering was done to a limited data set. These groups were projected onto UMAP analysis. We used ‘FindAllMarkers’ in Seurat to perform the differential gene expression analysis.

### 4.12 RNA velocity analysis

RNA velocity uses differences in the abundance of spliced and unspliced transcripts from the same gene to infer “directionality” in its regulation (i.e., whether it is in the process of up or downregulation).^16^ We used velocyto.R to estimate the differentiation process of CD8^+^T cells.

### 4.13 TCGA data analysis

We used the data downloaded from TCGA to evaluate the correlation between target genes and patient prognosis. We corrected the effect of T cell number in each group of the sample, referred to Chunhong Zheng et al.,^37^ the expression of target genes was divided by that of the geometric mean of CD3. We performed the statistical analysis via the ‘‘survival’’ R package. The survfit function was used to fit Survival curves and to evaluate the difference of the high and low gene expression groups.

### 4.14 Statistical analysis

We utilized unpaired Student's t‐tests to assess the significant differences between the two groups. When comparing multiple groups, the two‐way analysis of variance^38^ was used. Error bars indicate the SEM. *p*‐Values were represented as follows: *: *p* < .05, **: *p* < .01, ***: *p* < .001, and ****: *p* < .0001.

## CONFLICT OF INTEREST STATEMENT

The authors declare no conflict of interest.

## Supporting information

Supporting Information

Supporting Information

## Data Availability

The data supporting the conclusions of this paper have been provided in this paper and the TCGA database. In addition, all the data of the relevant dataset in this study can be obtained by contacting the corresponding author.
