# Supplementary material for: Single‐cell transcriptomics identify TNFRSF1B as a novel T‐cell exhaustion marker for ovarian cancer
Source: Clin Transl Med. 2023 Sep 15;13(9):e1416. doi: 10.1002/ctm2.1416 (PMC10502459; doi:10.1002/ctm2.1416)
Supplement: Supplementary file 1 — Supporting Information [file CTM2-13-e1416-s002.docx]

**Supplementary Figures**


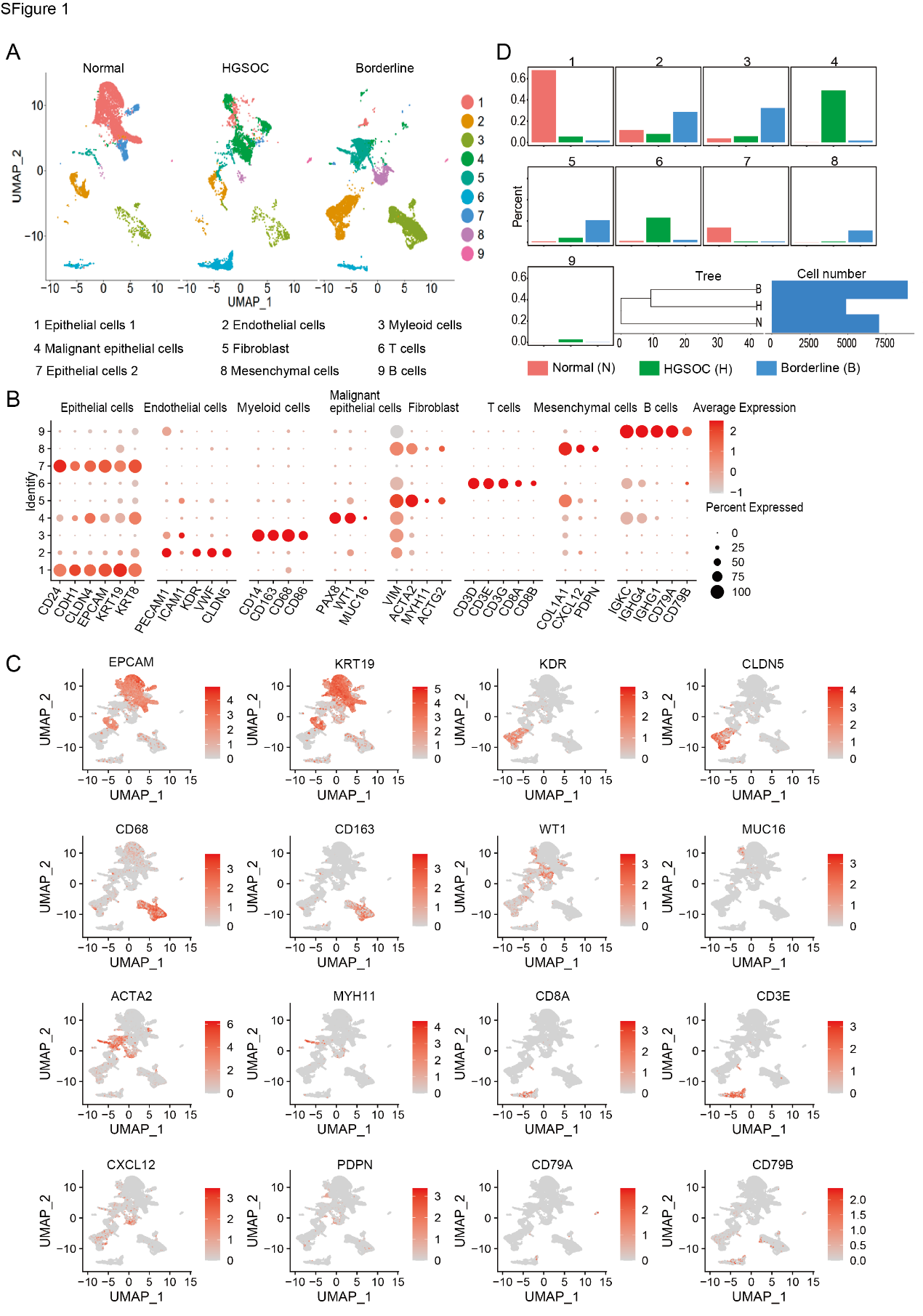


**Figure S1. Single-cell transcriptional profiling defines the TME of OC. (**A) UMAP plot of all the single cells, with each color coded for major cell types in normal ovary, borderline OC and HGSOC respectively. (B) Bubble chart depicting the differentially expressed genes and top marker genes of 9 major cell types. **(**C) Expression levels of specific markers for each cell type were plotted onto the UMAP. Color key from gray to red indicates relative expression levels from low to high. **(**D) Ratio of nine cell types in different tissues and the evolutionary tree showing the relationships of the three organizations

**
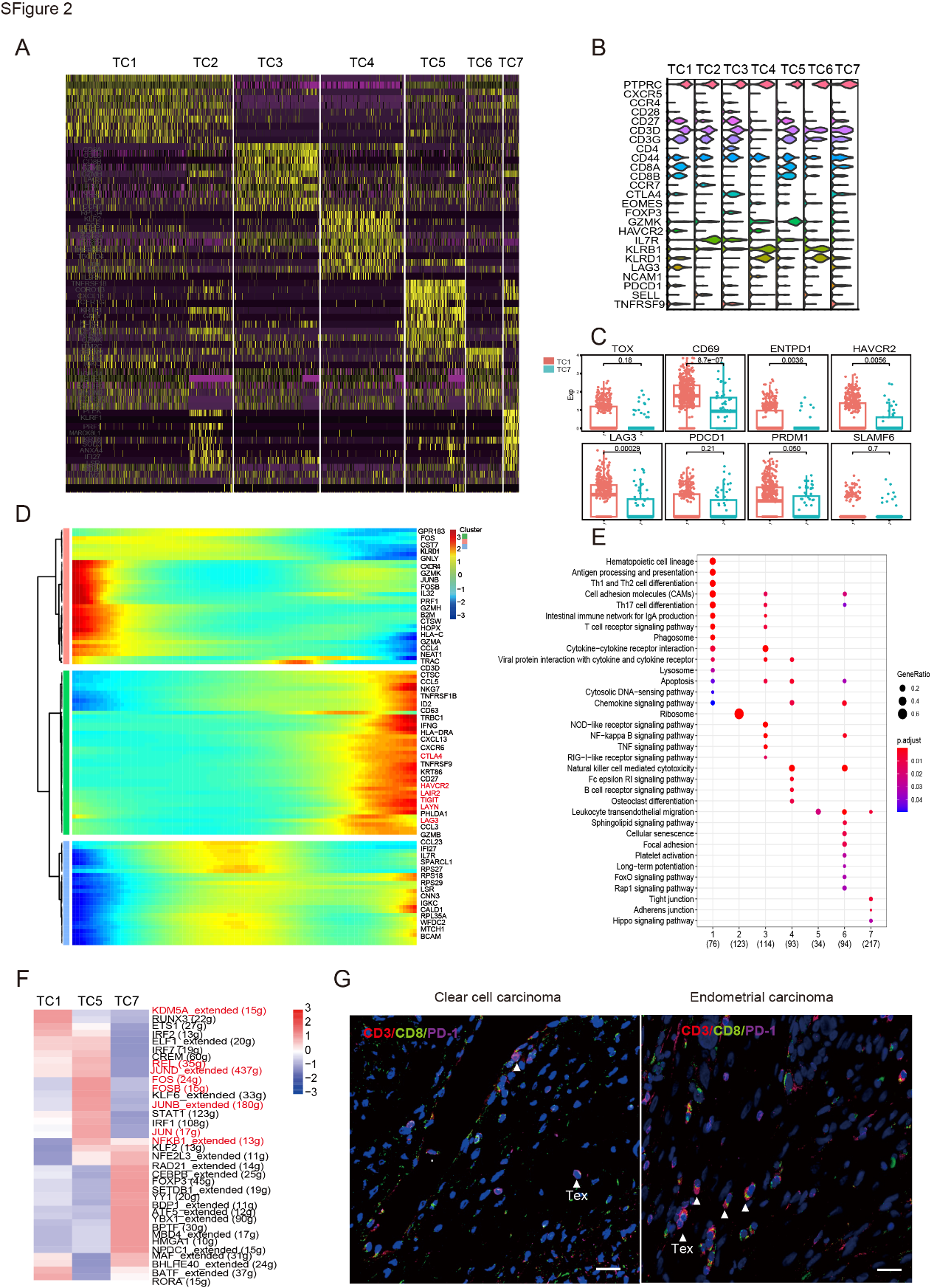
Figure S2.** **Differential gene expression signature between diverse T cell types.** (A) Heatmap displaying the differentially expressed genes in each cell types. (B) Volcano plot showing expression of representative markers in diverse cell types. **(**C) Heatmap showing differentially expressed genes rows along the pseudotime with the non-exhausted -to-exhausted T cells process. Color key from blue to red indicates relative expression levels from low to high. (D) The expression of representative exhausted markers in TC1 and TC7 subgroups. (E) GO analysis showing the biological function of diverse cell types. (F) Heatmap of differential regulons estimated by SCENIC between exhausted CD8^+^T cells (Tex1), exhausted CD8^+^T cells (Tex2) and non-exhausted CD8^+^T cells (Teff). **(**G) Multicolor immunofluorescence staining showing the presence of exhausted CD3^+^CD8^+^ T cells, in clear cell carcinoma and endometrial carcinoma patient, labeled with white arrow. The scale bar represents 50 μm.

**
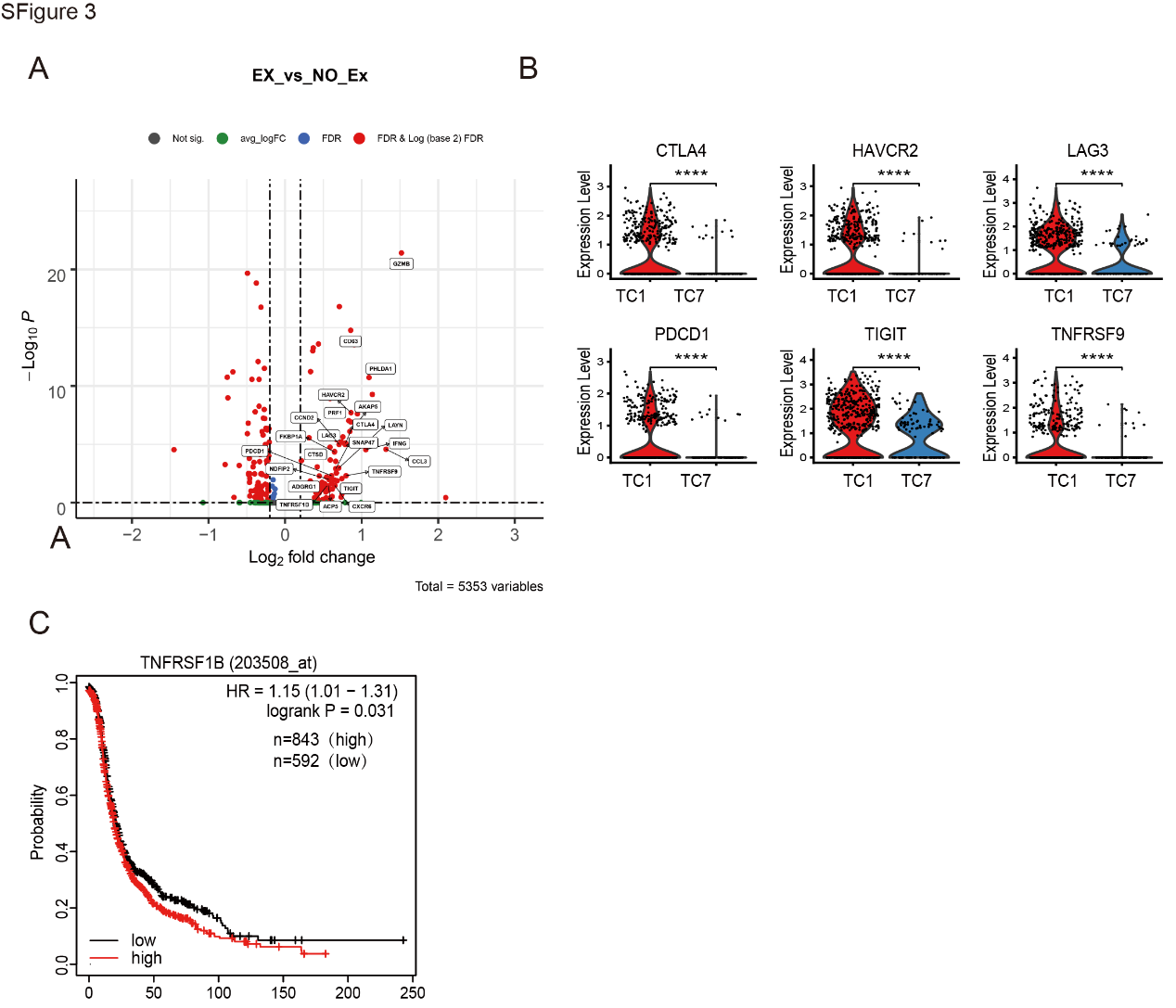
**

**Figure S3. Identification genes uniquely associated with OC-infiltrating exhausted CD8^+^ T cells. (**A) Volcano plot showing differentially expressed genes in exhausted and non-exhausted TILs. Each red dot denotes an individual gene passing our p value. (B) Violin plot demonstrating known exhausted genes expression level in exhausted and non-exhausted CD8^+^ T cells. ****p < 0.0001. (C) Kaplan-Meier curves displaying higher expression of TNFRSF1B predicted poor prognosis in ovarian cancer. p = 0.031, log rank tests, hazard ratio: 1.15 (1.01–1.31, 95% confidence interval [CI]).

**
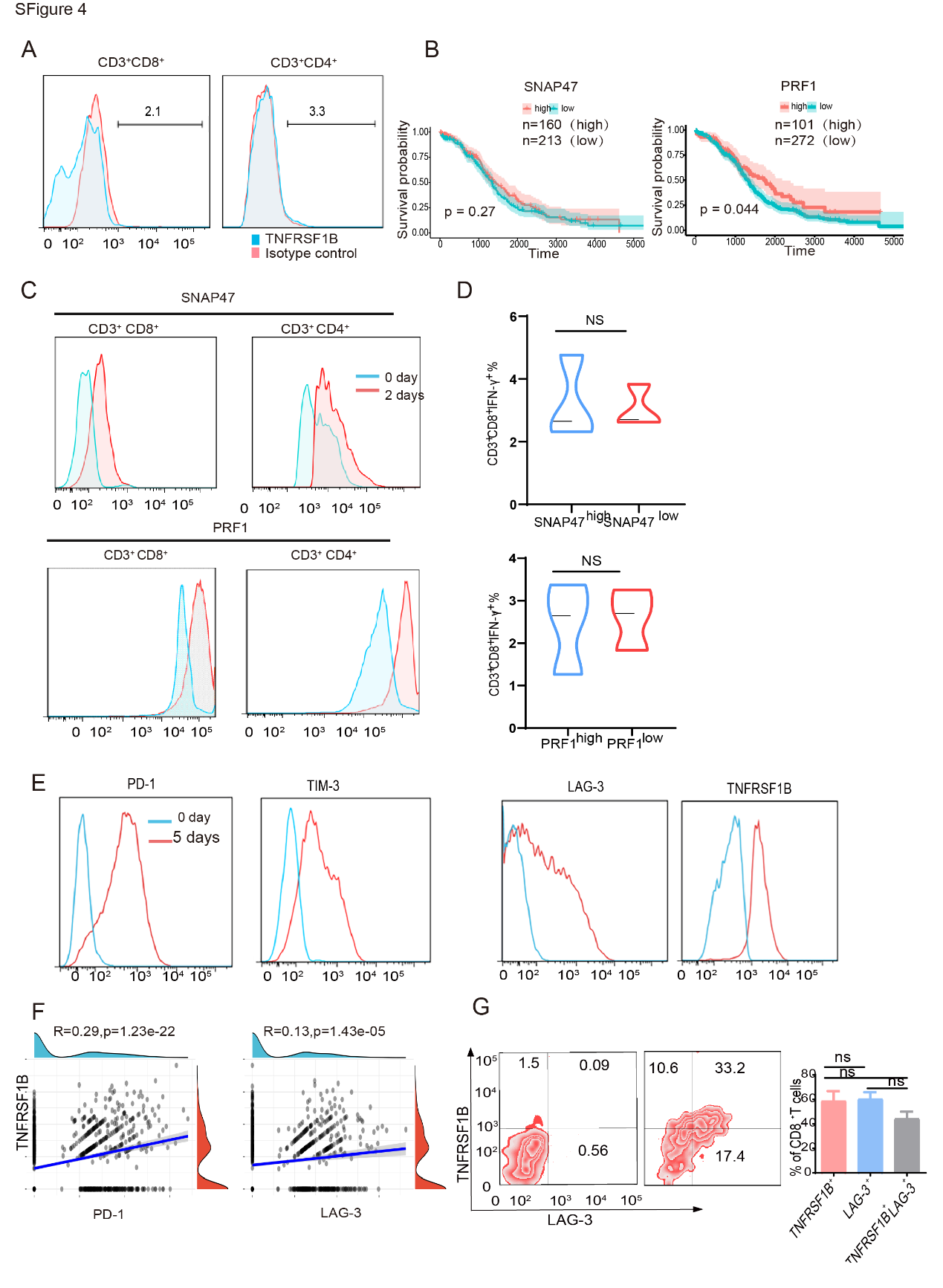
**

**Figure S4. The expression pattern of exhaustion-related genes in PBMC after activation.**

(A) Flow cytometry showing TNFRSF1B expression in the resting stage of CD3^+^CD8^+^T cells and CD3^+^CD4^+^ T cells isolated from Human PBMCs. (B) Survival curve comparing the high and low expression of SNAP47 and PRF1 based on cibersortx. (C) Human PBMCs were similarly stimulated as in Figure 4B for 2 days. Gated on CD8^+^T and CD4^+^T cells, SNAP47 and PRF1 expressions were determined by FACS. (D) Bar graph of human IFN-γ in the cell supernatant of sorted SNAP47^high^ and SNAP47^low^ CD8^+^T cells, PRF1^high^ and PRF1^low^ CD8^+^T cells. NS: no significant difference. Data represents mean ± SEM n=3. (E) Flow cytometry demonstrating TNFRSF1B and exhausted maker PD-1, TIM-3, and LAG-3 expression on CD8^+^T cells after stimulated in vitro with anti-CD3 and anti-CD28 monoclonal antibodies for 5 days. (F) he correlation between the expression of TNFRSF1B, PD-1 and LAG-3. (G) Flow cytometry plots showing TNFRSF1B and LAG-3 expression on CD8^+^T cells after stimulated in vitro with anti-CD3 and anti-CD28 monoclonal antibodies for 2 days.


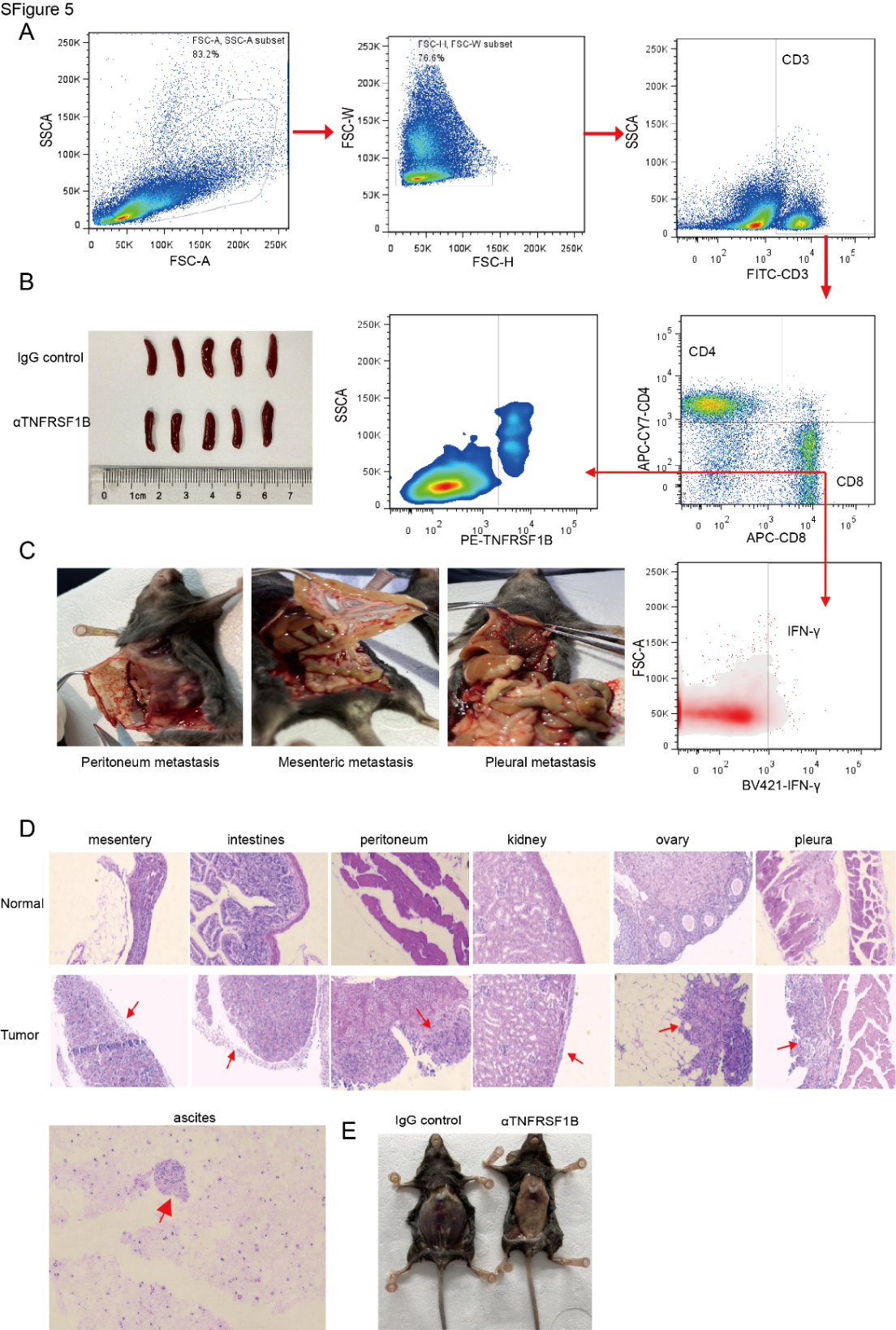


**Figure S5. Blockade of TNFRSF1B inhibits tumour growth in the ovarian cancer mouse model.**

(A) Flow Analysis flow chart. (B) Spleens were isolated and photographed from subcutaneous ID8-bearing mice after being treated with 200 mg anti-TNFRSF1B or isotype-matched control antibody IgG (n=5). (C) The mouse model of ovarian cancer metastasis was established (D) HE staining showed tumor metastasis in various organs. (E) The ascites of metastatic mice after different treatment at day 30.


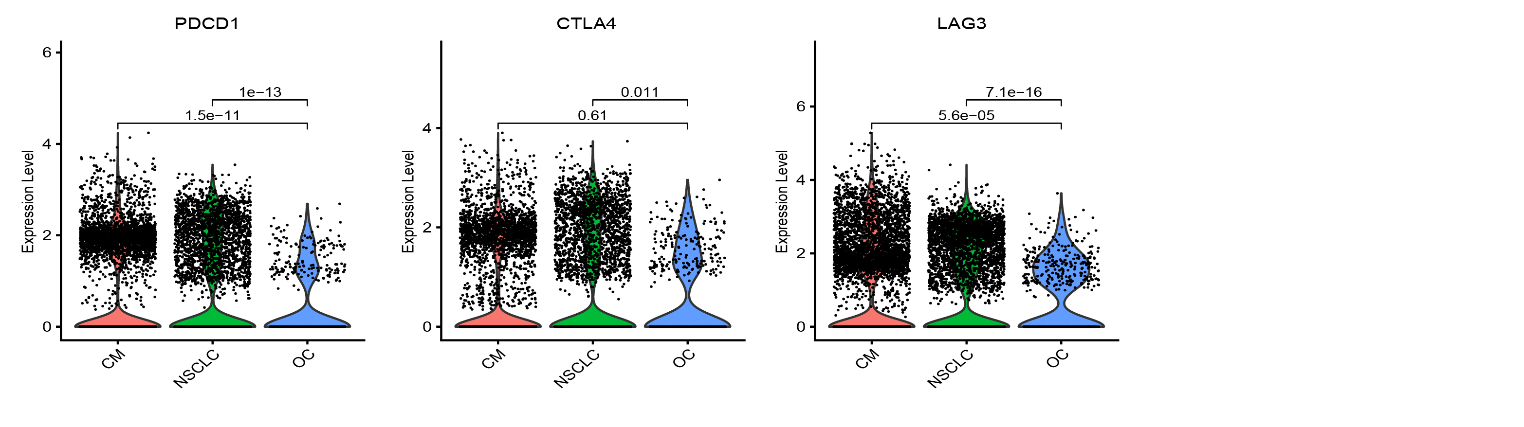


**Figure S6.** The expression level of exhausted markers in exhausted CD8^+^T cells of NSCLC, melanoma (CM) and ovarian cancer (OS).
